# Electrolyte Imbalance and Indirect Indicators of Dehydration in Temporary Agricultural Workers Exposed to Extreme Heat in the Mediterranean: An Observational Study on Environmental Health Risks

**DOI:** 10.3390/healthcare14010029

**Published:** 2025-12-22

**Authors:** Tania Cemeli, Glòria Tort-Nasarre, Judith Roca, Ana Lavedán-Santamaría, Carme Campoy, Laia Selva-Pareja, Jordi Vilaplana, Jordi Mateo, Anna Espart

**Affiliations:** 1 Department of Nursing and Physiotherapy, University of Lleida, C/Montserrat Roig 2, 25199 Lleida, Spain; tania.cemeli@udl.cat (T.C.); carme.campoy@udl.cat (C.C.); anna.espart@udl.cat (A.E.); 2 Health Education, Nursing, Sustainability and Innovation Research Group (GREISI), 25199 Lleida, Spain; 3 Development of Healthy and Sustainable Organizations and Territories (DOTSS), University of Lleida, Av. Estudi General 4, 25001 Lleida, Spain; 4 Department of Computer Science, University of Lleida, Av. Jaume II 69, 25001 Lleida, Spain; jordi.vilaplana@udl.cat (J.V.); jordi.mateo@udl.cat (J.M.)

**Keywords:** Mediterranean heat waves, heat stress, dehydration, electrolyte imbalance, agricultural workers, occupational health

## Abstract

**Highlights:**

**What are the main findings?**

- Agricultural workers in the Mediterranean are highly exposed to heat-related health risks.
- Heat exposure impacts cardiovascular and thermoregulatory functions.

**What are the implications of the main findings?**

- Continuous monitoring of physiological signs can help prevent heat-related disorders.
- Adaptive and preventive strategies are essential to protect vulnerable outdoor laborers.

**Abstract:**

**Background:** Climate change is intensifying extreme heat exposure in Mediterranean agricultural systems. Migrant workers engaged in outdoor fieldwork are a highly vulnerable population with limited access to resources. Crucially, there is a notable lack of data on how heat affects these workers in this specific region. **Objective:** This study aimed to analyze the physiological effects of high-temperature exposure by quantifying and correlating indirect indicators of dehydration and electrolyte imbalance (sodium and potassium losses, sweat, body weight, and blood pressure). **Methods:** An observational study was conducted over nine consecutive days involving ten agricultural participants, yielding 90 observations. Measurements of body weight, heart rate, blood pressure, skin temperature, sweat loss, and sodium and potassium concentrations were taken before, during, and after daily field activity. **Results:** Results showed considerable interindividual variability in thermophysiological responses. Participants lost an average of 0.8 kg (range –9.1 to +3.6 kg) and produced 3.91 L of sweat (range 1.9–6.4 L), with sodium and potassium losses of 4932 mg and 646 mg, respectively. Sweat loss correlated with sodium (r = 0.414, *p* = 0.001) and potassium (r = 0.791, *p* < 0.001), and diastolic blood pressure was moderately associated with weight loss (r = 0.576, *p* = 0.016). **Conclusions:** Sweat loss was the main driver of electrolyte depletion, with marked interindividual variability. Monitoring sweat-related indicators and diastolic blood pressure could help detect dehydration risk in agricultural workers exposed to extreme heat. Targeted hydration strategies and occupational health education are essential to mitigate these risks.

## 1. Introduction

Climate change is one of the greatest challenges humanity faces today, characterized by long-term shifts in climatic patterns and an increase in extreme events such as heavy precipitation, prolonged droughts, and—particularly—intensifying heat waves. These changes are largely attributed to human activities, especially fossil fuel combustion, deforestation, and intensive land use, which continue to drive current warming trends [1,2].

The Mediterranean region is particularly affected by droughts, wildfires, and extreme heat waves. Specifically, extreme heat waves are becoming increasingly common and frequent during the climatological summer months (i.e., June–September) in inland regions. In Spain, heat waves have become progressively more frequent and intense over the past decade. Notable records include the longest heat wave in 2015 (26 consecutive days), the highest number of episodes in a single season in 2017 (seven events), and the largest geographical area affected by a single heat wave in 2022, covering 377,000 km^2^ [3,4]. These increasingly severe heat waves generate substantial heat stress that affects ecosystems and human health. For exposed populations, they contribute to acute conditions—such as heat stroke, exhaustion and muscle cramps—and to longer-term consequences including chronic dehydration and disturbances in electrolyte balance [5,6].

Agricultural workers, who spend many hours under the effects of extreme temperatures, are particularly vulnerable [7]. Most of these workers are migrants, facing precarious working conditions and lacking access to essential resources, which increases their susceptibility to the adverse effects of heat stress and dehydration. This vulnerability is further exacerbated by limited access to shade, adequate cooling systems, and water sources, heightening their risk of health problems related to heat stress and dehydration. Additionally, language barriers and migratory status hinder their access to essential resources, increasing their risk of health issues associated with heat stress [8,9]. Different authors have also reported not only these alterations in agricultural workers but also serious health problems such as chronic kidney disease, cardiovascular diseases, and metabolic disorders [10,11,12].

Early detection of dehydration and indirect dehydration indicators (IID) is crucial for assessing health risks in agricultural workers. Heat stress greatly increases sweating and, with it, the loss of key electrolytes such as sodium and potassium. Because these electrolytes are essential for blood pressure regulation, muscle function, and thermoregulation, their imbalance can rapidly worsen heat-related strain and elevate the risk of serious illness. Therefore, examining electrolyte imbalance is fundamental to understanding workers’ physiological vulnerability during high-temperature exposure. In addition, the demanding and changing working conditions of many agricultural laborers—often migrant workers adapting to different climates and workloads—may further intensify their susceptibility to dehydration and electrolyte loss [5,12,13].

Despite studies conducted in other climatic regions, there is a notable and critical lack of data on how heat waves and high-temperature exposure affect migrant agricultural workers in Spain. This absence of empirical evidence is especially alarming given the Mediterranean region’s increasing vulnerability to climate change and the high reliance of Spanish agriculture on temporary and often migrant labor [5,14]. These workers perform physically demanding tasks under direct sun exposure, often with limited access to preventive measures, making this data gap particularly urgent to address.

Understanding the physiological impacts of extreme heat—particularly in terms of IID and electrolyte imbalance (e.g., sodium and potassium loss)—is crucial for informing targeted occupational health strategies. Such data are essential for the development of evidence-based public health policies that protect this vulnerable group, improve their working and living conditions, and ensure their safety and overall well-being in the context of a changing climate.

The aim of the study was to analyze the effects of outdoor agricultural work under high-temperature conditions on IID, specifically focusing on sodium and potassium loss and their relationship with sweat loss and other physiological variables, while accounting for individual variability and characterizing sweat composition and fluid loss using a real-time wearable monitoring device.

## 2. Materials and Methods

### 2.1. Study Design and Participants

An observational design was employed to systematically record a set of data from the participants throughout their workday. The study population was a convenience sample of ten temporary agricultural workers (n = 10) participating in the summer fruit-picking campaign in the region of Lleida, Spain. Participants were recruited in July 2023 through direct collaboration with the farm owner and a local non-governmental organization that provides support to migrant workers. This agricultural area is characterized by a semi-continental Mediterranean climate. At the time of data collection, workers had already begun the pear-harvesting season, indicating they were heat-acclimated.

Inclusion criteria required participants to be (1) adults (over 18 years old); (2) actively performing outdoor agricultural tasks for the entirety of the work shift; (3) able to communicate effectively for consent and interviews; and (4) free from known chronic cardiovascular or renal diseases. Exclusion criteria included having any acute illness or injury at the time of the study or being pregnant. All eligible workers who expressed interest were fully informed about the study protocol and provided written consent.

To conduct the study, data were collected from participants at the beginning of the study, as well as during all workdays throughout its duration. The data were recorded in real time before, during, and immediately after the workday.

Although a convenience sample of 10 participants was utilized for this observational study, this sample size was deemed appropriate due to the intensive nature of the data collection. The protocol involved multiple real-time physiological measurements (including weight, heart rate, blood pressure, skin temperature, and sweat/electrolyte loss) taken before, during, and after the work shift over nine consecutive days. This methodology resulted in a total of 90 observations (10 participants × 9 days) for the key variables related to hydration and heat stress. This high volume of within-subject observations was critical for exploring interindividual variability and establishing the statistically significant correlations reported in Section 3, partially compensating for the small number of participants.

### 2.2. Variables and Data Collection

An ad hoc questionnaire was specifically developed for this study and used to record the variables of interest. The questionnaire collected sociodemographic information at the beginning of the study, along with health-related variables.

Sociodemographic variables recorded included age, sex, education level, medical history or medication use, physical activity habits, usual eating habits (frequency of consumption of fruits, vegetables, water, and other liquids), amount of water consumed during workdays, and average hours of sleep during the night.

Data collection took place during the afternoon hours, when environmental temperatures tend to be highest -i.e., 2:00 p.m. to 6:00 p.m. Data collection was carried out between 15 and 30 July 2024, over nine consecutive days during the same afternoon time. All participants provided written informed consent prior to their inclusion in the study. Health variables included: weight, heart rate, systolic and diastolic blood pressure, skin temperature, sweat loss, sodium loss, and potassium loss. To measure health variables, weight was assessed using a scale, blood pressure and heart rate were monitored with an electronic blood pressure device, and the reusable hDrop device (https://hdroptech.com/ (accessed on 12 May 2024)) was employed to measure skin surface temperature, sweat, sodium, and potassium loss. Selected for its capability to provide continuous, non-invasive, real-time estimations of sweat rate and electrolyte losses directly from the skin surface, the device was affixed to the participants’ arms before the commencement of the afternoon work shift (2:00 p.m.) and remained in place until the end of the shift (6:00 p.m.), continuously recording real-time data. All devices used in the study, including the scale, blood pressure monitor, and hDrop device, were calibrated according to the manufacturer’s instructions prior to each use to ensure accurate and reliable measurements. Participants had access to water ad libitum during the four-hour work period, and the amount consumed was recorded. No electrolyte-containing beverages or food were consumed during this period. Additionally, ambient temperature and humidity were continuously monitored. These data were obtained from the nearest national weather station, located 5 km from the participants’ location.

Data collection was conducted by two external health professionals trained to ensure standardized and accurate measurement of all variables. The recorded data were subsequently entered into a database for analysis. Written informed consent was obtained from all participants before study inclusion. For each variable, values were averaged across participants and all measurements obtained during the 9-day observation period. Results are expressed as mean ± SD.

Body weight loss was used as an indirect indicator of physiological responses related to IID, categorized as: (i) 1–3% (mild), (ii) 3–5% (moderate), and (iii) >5% (severe). In addition, according to suggested data on sweat electrolyte composition [15], we considered the potential for electrolyte deficits. Sodium losses were categorized as (i) 0.5–1 g Na^+^/L (mild potential deficit), (ii) 1–1.5 g Na^+^/L (moderate), and (iii) >1.5 g Na^+^/L (severe). Potassium losses were categorized as (i) 160–200 mg K^+^/L (mild), (ii) 200–280 mg K^+^/L (moderate), and (iii) >280 mg K^+^/L (severe) [16,17].

### 2.3. Data Analysis

Descriptive statistics were utilized to summarize demographic characteristics, physiological measurements, and questionnaire responses. Correlation analysis was employed to examine the relationships between physiological parameters, meteorological conditions, and work intensity. To identify factors associated with heat stress and IID, linear regression and mixed-effects models were employed. Specifically, given the repeated, longitudinal measurements, the mixed-effects model incorporated a random identity factor for each participant. This approach allowed for the control of individual variations and the precise assessment of covariates’ effects on the physiological variables. Missing data were handled using multiple imputation methods to minimize bias. IBM-SPSS Statistics version 28 (IBM SPSS Statistics for Windows, Version 28.0. IBM Corp, Armonk, NY, USA) and RStudio version 4.3.1 (R Core Team) (packages “nlme” and “ggplot2”) were used for the analysis.

## 3. Results

**Figure 1:**
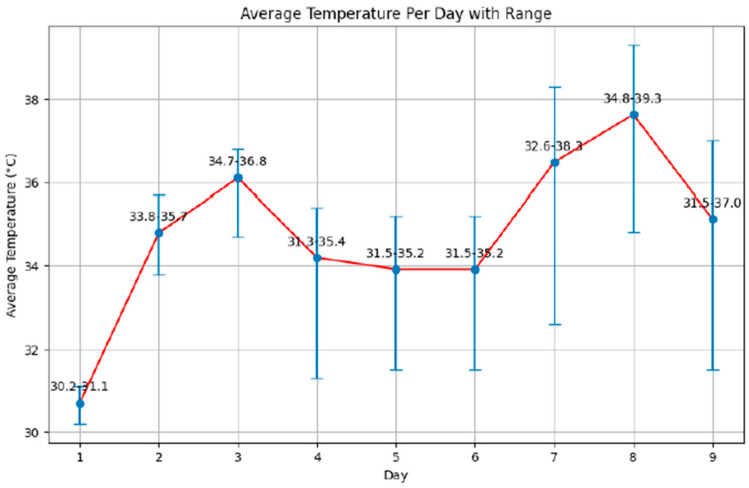
Evolution of ambient temperature registered during the workday. Blue lines represent the temperature range measured during the four daily observation hours, while the red line indicates the mean temperature recorded for each day.

The study was conducted with a group of 10 agricultural workers, of Senegalese and Gambian origin, from a farm, selected to evaluate the impact of heat stress during heat waves. Throughout the study, 90 observations were made, covering nine key variables directly related to hydration and heat stress. All participants were men, with an average age of 45.80 years (SD = 14.88), and an age range from 22 to 65 years. They drank an average of 1.5 L of water during the four hours in which the measurements were taken, and slept an average of 6.4 h (SD = 1.35). Each participant recorded an 8 h workday, exposed to the outdoors under direct sunlight or semi-shaded conditions. During the workday, they all took a two-hour break between 12:00 p.m. and 2:00 p.m. (solar time). The ambient average temperatures during the data recording days ranged from 30.2 °C to 39.3 °C, with day seven having the greatest temperature range, showing a difference of 5.7 °C, and day one having the smallest range (0.9 °C) (Figure 1).

### 3.1. Main Physiological Health Characteristics of Participants

While some variables exhibited a normal distribution (*p* > 0.05), others—such as heart rate, body weight, diastolic blood pressure, sodium concentration, and potassium loss—showed markedly skewed distributions (*p* < 0.05), indicating heterogeneous physiological responses among participants. Differences in skin temperature and sweat-related parameters demonstrated substantial variability, reflecting individual differences in thermoregulation and electrolyte balance during the study. Mean sweat loss was 3.91 L (range: 1.9–6.4 L), with corresponding sodium and potassium losses of 4932 mg and 646 mg, respectively. All values represent changes between post-work and pre-work measurements. The weight difference range includes one extreme value (−9.1 kg), likely attributable to measurement error, which was retained to ensure transparency (Table 1).

### 3.2. Relationship Between Physiological Health Variables and Temperature

**Figure 2:**
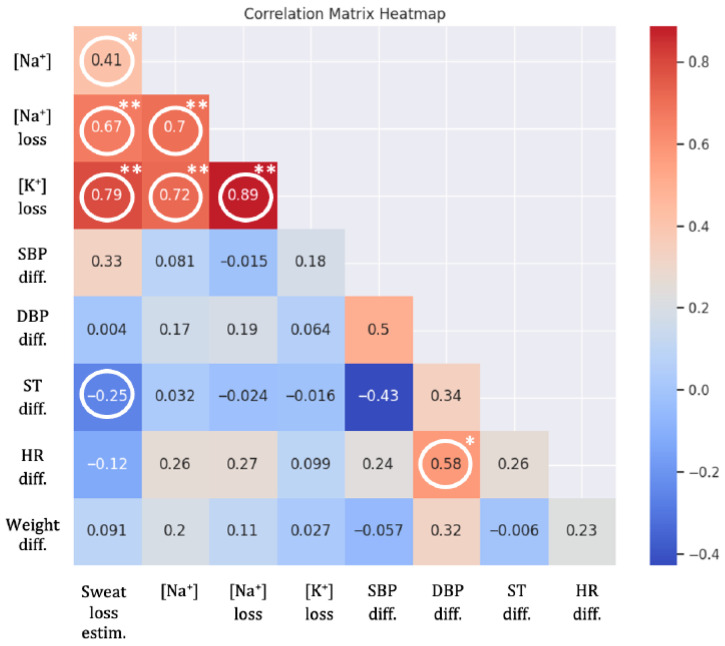
Correlation heatmap between temperature and physiological variables. The circled values indicate a statistically significant relationship between the two variables. *: moderate significant correlation; **: highly significant correlation. [Na+]: sodium concentration; [Na+] loss: loss of sodium concentration; [K+] loss: loss of potassium concentration; SBP diff: systolic blood pressure difference; DBP diff: diastolic blood pressure difference; ST diff: skin temperature difference; HR diff: heart rate difference; Weight diff: weight difference; Sweat loss estim.: sweat loss estimation.

In relation to the variables related to weight loss, sodium concentration, and potassium loss, some statistically significant correlations were observed. Specifically, for sweat loss, significant correlations were found between this variable and sodium concentration (r = 0.414, *p*-value = 0.001) and potassium concentration (r = 0.791, *p*-value < 0.001). Additionally, skin temperature and sweat loss estimation were weakly correlated (r = –0.254, *p* = 0.046), indicating a weak inverse relationship between sweating and skin surface temperature. Sodium loss showed strong correlations with potassium loss (r = 0.885, *p*-value < 0.001) and sodium concentration (r = 0.703, *p* < 0.001), but had weak correlations with blood pressure, heart rate, and weight. However, the relationships between these electrolytes and systolic/diastolic blood pressure, heart rate, and body weight were not significant, suggesting no association among these variables (Figure 2).

Disparities were observed in the variables of differences in systolic and diastolic blood pressure, skin temperature, and heart rate. Regarding the systolic blood pressure (SBP), a moderate and significant correlation was found with the difference in diastolic blood pressure (DBP) (r = 0.499, *p*-value = 0.035), indicating that changes in SBP and DBP occurred together. Conversely, DBP showed a moderate and significant correlation with weight difference (r = 0.576, *p*-value = 0.016), suggesting a relationship between weight loss and blood pressure variation in response to heat exposure (Figure 2). Specifically, SBP generally decreased during the work session, with a range from −35.0 to 6.0 mmHg, while DBP exhibited a wider range of change, from −21.0 to 61.0 mmHg. These variations did not follow a consistent trend over the nine days of observation, indicating individual variability in cardiovascular response to heat. A positive correlation was observed between body weight loss and diastolic blood pressure, indicating that participants who lost more weight tended to show lower diastolic values.

Finally, the variables of skin temperature and heart rate differences did not show a significant relationship with any of the analyzed variables.

### 3.3. Levels of Indirect Indicators of Dehydration in Participants

The recording of variables before and after the workday, as well as those recorded by the hDrop system, allowed for the description of the degree of IID due to weight loss (sweat), decrease in sodium concentration, and decrease in potassium concentration. Based on 90 observations, IID due to weight loss (sweat loss) was determined in 49.2% (n = 29) of the cases, moderate in 72.6% (n = 45) due to a decrease in sodium concentration, and absent in 50% (n = 28) in relation to the decrease in potassium concentration. Thus, the levels of IID varied according to the observed variable (Table 2), highlighting the need for hydration strategies that include electrolyte replacement.

### 3.4. Analysis of Factors Associated with Physiological Losses

For the analysis of factors associated with sweat, sodium, potassium, and weight loss in workers, a mixed-effects model was used. Since each participant could present individual variations that might influence the responses of physiological variables, the applied model incorporated a random identity factor, thus allowing for the control of these individual differences and the precise adjustment of the effects of the covariates. Sodium loss exhibited a positive and highly significant relationship with weight difference (T = 7.02; *p*-value < 0.001) and skin temperature (T = 7.07; *p*-value < 0.001) (Table 3).

On the other hand, neither the constant nor the variables day and differences in skin temperature reached statistical significance. In contrast, two pertinent factors were identified: the difference in diastolic blood pressure (T = 2.21; *p*-value = 0.031) and the estimated sweat loss (T = 5.89; *p*-value < 0.001) (Table 4).

Finally, the mixed model results showed that potassium loss was significantly influenced by sweat loss estimation (T = 7.02, *p* < 0.001), which emerged as the strongest predictor, and by sodium loss (T = 5.96, *p* < 0.001). Additionally, a significant inverse relationship was observed with diastolic blood pressure difference (T = –2.47, *p* = 0.016) (Table 5).

## 4. Discussion

The findings of this study underscore the significant impact of heat stress on agricultural workers, particularly migrants working under extreme temperature conditions. Indirect indicators of dehydration (IID), evidenced by weight loss and electrolyte depletion, emerged as a prevalent issue, with sweat loss identified as the main driver of sodium and potassium imbalance [10,11,12].

### 4.1. Impact of Temperature on Physiological Health Characteristics

Despite the limited number of participants, the variability observed in skin temperature and electrolyte losses suggests that individual factors—such as acclimatization and physiological capacity—may strongly influence susceptibility to heat stress. This heterogeneity has practical implications: workers with lower sweating efficiency or inadequate electrolyte replacement could face higher risks of dehydration and cardiovascular strain. The strong link between sweat loss and electrolyte depletion reinforces the need for preventive strategies that go beyond water intake, incorporating electrolyte-enriched hydration and education on heat stress management to protect vulnerable agricultural populations. This finding is pivotal for developing rehydration strategies that not only replenish lost water but also restore essential electrolytes. Although this assertion has been corroborated by various authors [18,19], it is essential for the supervisors of these workers to consider it. Providing electrolyte-enhanced hydration may help reduce dehydration-related risks and support thermoregulation during heat exposure, as suggested by previous studies.

Regarding the impact of heat on the cardiovascular response of the participants, the differences in systolic and diastolic blood pressure showed significant variability. This could indicate that heat exposure affects the cardiovascular response of workers differently, which may have implications for health management in hot work environments. It is also interesting to note that the moderate correlation between the difference in diastolic blood pressure and weight loss could indicate that fluid loss affects blood pressure. To the best of our knowledge, there are few studies that correlate the decrease in diastolic blood pressure and weight loss as effects of sweating under extreme heat working conditions.

However, there is evidence of a correlation between blood pressure and weight loss, whether associated with a decrease in body mass index or as a consequence of an acute post-exercise effect [20]. These results indicate a potential avenue for future research on the interplay between temperature, weight, sweat loss, and diastolic blood pressure in outdoor workers. Therefore, monitoring cardiovascular parameters such as blood pressure and heart rate may be beneficial in certain contexts, particularly for identifying signs of heat-related stress. However, such monitoring should consider the practical limitations of field settings and the influence of individual factors such as hydration status, physical exertion, heat acclimation, and fitness level.

Sweating is a primary thermoregulatory mechanism that typically increases with rising core skin temperature [21]. In this study, a weak inverse correlation was observed between skin surface temperature and sweat rate. However, this does not imply a causal relationship. It is possible that as IID progresses, hormonal responses such as aldosterone and catecholamine release may reduce sweating to conserve body water and plasma volume. This physiological adaptation, while aiming to preserve circulatory stability, may inadvertently contribute to a rise in skin temperature as the individual continues to work under heat stress [22]. This interpretation should be approached with caution, as multiple physiological factors—including hydration status, heat acclimatization, and individual variability—can influence both sweating and temperature regulation.

### 4.2. Indirect Indicators of Dehydration and Electrolyte Loss

Extreme heat events, exacerbated by climate change, are increasingly common in Mediterranean regions [23], leading to significant IID among agricultural workers. This highlights the urgent need for targeted interventions to mitigate the health risks associated with prolonged exposure to high temperatures.

Our results further confirm the prevalence of IID among agricultural workers exposed to high temperatures. Nearly half of the participants experienced IID due to weight loss, and 72.6% exhibited moderate potential electrolyte deficits due to sodium loss in sweat. These findings highlight the substantial risk faced by this vulnerable population and underscore the need for specific interventions to mitigate the effects of heat stress. This is consistent with previous studies that have identified agricultural workers as particularly susceptible to heat-related health issues [10,11,12,16]. Additionally, our study provides new insights into the correlation between sodium and potassium loss, as well as the role of fluctuations in skin temperature and blood pressure in IID patterns. Furthermore, the observed variability in IID levels suggests that individual factors, such as thermoregulation capacity or heat acclimatization, may influence susceptibility to heat stress.

While the correlation between sweat loss and electrolyte depletion is physiologically expected, the novelty of this study lies in documenting these patterns in real-world agricultural settings under Mediterranean heat conditions, introducing indirect indicators of dehydration (IID) as a practical monitoring framework, and highlighting significant interindividual variability in thermophysiological responses. Additionally, the observed association between diastolic blood pressure and weight loss offers a potential field marker for dehydration risk, which has not been widely explored in occupational health literature.

Hydration practices also play a critical role. During the afternoon work shift, participants’ average water intake was approximately 375 mL/h (1500 mL over 4 h). Although this intake provides some hydration, it may be insufficient under high-temperature conditions and physically demanding labor. García-Trabanino et al. [17] have suggested that water intake of up to 800 mL/h may be necessary to prevent dehydration and reduce the risk of heat-related illnesses, including potential renal impairment.

Similarly, other researchers, such as Abasilim et al. [16], have documented high rates of dehydration among agricultural workers, attributing these findings to demanding working conditions and prolonged heat exposure. In our study, although data were only recorded for 4 h (half of the workday), these measurements were scheduled during the afternoon hours (2:00 p.m. to 6:00 p.m.), as defined in the study protocol, which coincided with the period of highest ambient temperatures. This timing was chosen a priori to capture the physiological responses under the most thermally stressful conditions, not based on daily temperature fluctuations.

As Wagoner et al. [7] have indicated in their study, agricultural workers, especially migrants, are particularly vulnerable to heat stress due to their working conditions and lack of access to essential resources. This aligns with our findings, reinforcing the idea that even short periods of exposure to high temperatures can lead to significant dehydration, as reflected by IID. These results highlight the critical need for continuous monitoring and timely interventions to prevent dehydration and its associated health risks, even during shorter periods of heat exposure.

Regular monitoring of electrolytes is crucial for the early detection and prevention of health risks associated with heat stress. Implementing appropriate interventions, such as providing electrolyte replacement solutions, ensuring access to clean drinking water, and educating agricultural workers or other outdoor workers on the signs and symptoms of dehydration, can significantly mitigate these risks. Furthermore, previous studies demonstrated the effectiveness of establishing educational programs to help workers recognize the signs of IID and other heat-related illnesses, thereby preventing related health issues [24,25]. These findings underscore the importance of comprehensive health and safety strategies that include both monitoring and education to protect vulnerable worker populations from the adverse effects of heat exposure.

Future research should prioritize the development and evaluation of interventions aimed at mitigating heat stress and dehydration among agricultural workers. These interventions could encompass the provision of adequate hydration resources, implementation of cooling systems, and education on recognizing early signs of dehydration [26]. Furthermore, longitudinal studies with larger sample sizes are necessary to yield more comprehensive data on the long-term health impacts of heat stress within this population.

### 4.3. Practical Implications

Strategies to reduce dehydration, as reflected by IID, such as frequent breaks, access to drinking water, and hydration education, are essential for protecting the health of workers exposed to hot conditions. The study results highlight the importance of implementing these preventive measures, which have proven effective in other contexts, such as Nicaragua [24]. In this regard, implementing similar strategies tailored to the reality of workers in Mediterranean regions could have a positive impact, helping to reduce dehydration-related risks—as evidenced by indirect indicators—and improve the health and well-being of agricultural workers.

Employers and policymakers should prioritize the health and safety of agricultural workers by improving working conditions and providing the necessary resources to prevent heat-related illnesses. However, current strategies present certain limitations that may hinder their effective application in the workplace. Factors such as the pressure to meet production targets or long working hours can reduce breaks or diminish the availability of drinking water. Additionally, the lack of knowledge about the effects of dehydration and the importance of proper hydration may lead workers to not prioritize these measures. This creates a gap between the suggested interventions and their practical application, as workers might not recognize the need to hydrate adequately during the workday.

To overcome these barriers, it would be essential to develop specific training programs tailored to the conditions and realities of agricultural workers. The training should include clear information about the risks of dehydration and preventive measures, such as the importance of breaks and continuous hydration. Additionally, these educational initiatives should include a practical component, ensuring that workers can apply the acquired knowledge in their daily routines. Collaboration with employers to ensure that these measures can be effectively implemented is key to overcoming work-related pressure barriers and improving workers’ health.

### 4.4. Limitations

The study has limitations. The convenience sample of 10 migrant seasonal agricultural workers from a specific agricultural field may limit the generalizability of the findings. The structured questionnaire, while comprehensive, relies on self-reported data, which can be subject to recall bias. The observational design may not capture variations in heat stress and IID throughout the peak summer heat period. Physiological measurements and pre- and post-workday weight measurements provided valuable data, but may have some inconsistencies in accuracy and usage due to potential environmental factors. Ambient conditions recorded by a national weather station may not reflect the microclimate experienced by workers. The small sample size limits the statistical power of the results. Although individual water intake was recorded during the study, this information was not used to adjust the calculations of sweat rate or total sweat loss. As a result, the values presented may slightly overestimate actual sweat loss, which should be considered when interpreting the results. Overall, the study provides valuable insights into the effects of heat stress and IID among seasonal agricultural workers, but the limitations related to sample size, data collection methods, and potential biases should be considered when interpreting the results. Furthermore, the study included only male participants; thus, the findings cannot be directly extrapolated to female agricultural workers, who might exhibit different physiological responses and vulnerabilities to heat stress. All temperature measurements in this study were obtained using the hDrop device, which records skin surface temperature. This method does not provide core skin body temperature values. While skin temperature can reflect thermal stress, it may not accurately represent internal skin temperature, which is a limitation of this approach. Another limitation is the use of the hDrop device, which lacks external validation for skin temperature or electrolyte losses.

Finally, multiple regression analyses were performed to explore potential associations between physiological parameters and markers of IID. However, given the small sample size (n = 10), these analyses are exploratory in nature and should be interpreted with caution, as they are not adequately powered to establish definitive causal relationships.

## 5. Conclusions

This study reveals that indirect indicators of dehydration (IID) are prevalent among agricultural workers exposed to high temperatures, with nearly half of the participants showing signs of fluid loss based on weight changes and 72.6% presenting moderate levels of IID related to sodium loss. Sodium and potassium losses were significantly correlated, primarily driven by total sweat volume, as would be expected. Additionally, diastolic blood pressure is also influenced by IID levels, which could open new avenues for future research. The variability in IID levels suggests that individual factors, such as thermoregulation capacity or heat acclimatization, may influence susceptibility to heat stress. The results underscore the substantial risk faced by this vulnerable population, highlighting the need for specific interventions to mitigate the effects of heat stress. Strategies such as frequent breaks, access to drinking water, and hydration education are essential for protecting the health of workers exposed to hot conditions.

Further studies are needed to confirm these findings and to better understand the implications of the results obtained in this study.

## Figures and Tables

**Table 1:** Exploratory and descriptive analysis of physiological variables.

Variable	Rank (Min/Max)	Mean	SD	IQR	*p*-Value
Heart rate (bpm) diff.	−20.00/34.0	−8.50	13.5	17.8	**0.020**
Weight (Kg) diff.	−9.1/3.6	−0.80	1.7	0.8	**<0.001**
Systolic blood pressure (mmHg) diff.	−35.0/6.0	0.00	12.2	23.0	0.185
Diastolic blood pressure (mmHg) diff.	−21.0/61.0	0.00	17.5	15.7	**0.002**
Skin temperature (°C) diff.	1.90/16.8	9.40	2.6	2.4	0.062
Sweat loss (total L)	1.9/6.4	3.91	1.0	1.7	0.145
Sweat rate (L/h)	0.4/1.4	0.96	0.2	0.3	0.336
Sodium concentration (mg/L)	620.1/4423.0	1262.00	470.3	328.7	**<0.001**
Sodium loss (mg)	1009.0/9669.0	4932.00	2031.1	2752.2	0.200
Potassium loss (mg)	150.8/1645.0	646.00	282.7	380.9	**0.003**

SD: standard deviation; IQR: interquartile range; bpm: beats per minute; mmHg: millimeters of mercury. *p* indicates the significance level from the Shapiro–Wilk test for normality. Statistically significant *p*-values are shown in bold. Values represent differences between post-work and pre-work measurements. The weight difference range includes one extreme value (−9.1 kg), likely due to measurement error, but retained for transparency.

**Table 2:** Observed percentages and frequencies of IID according to weight loss (sweat), sodium, and potassium.

IID Due to		Percentage (n)
Weight loss (sweat)	No	44.1% (26)
	Mild	49.2% (29)
	Moderate	3.4% (2)
	Severe	3.4% (2)
Sodium loss	No	0
	Mild	11.3% (7)
	Moderate	72.6% (45)
	Severe	16.1% (10)
Potassium loss	No	50.0% (28)
	Mild	33.9% (19)
	Moderate-Severe	16.1% (9)

IID: indirect indicators of dehydration.

**Table 3:** Coefficients of the mixed model adjusted by REML for sweat loss.

Fixed Effects	Estimator	SE	DF	T	*p*-Value
Constant	−5.510	2.486	72	−2.22	**0.030**
Day	−0.042	0.022	72	−1.93	**0.057**
ST diff.	−0.042	0.019	72	−2.23	**0.029**
SBP diff.	−0.003	0.012	72	−0.25	0.804
DBP diff.	0.012	0.009	72	1.39	0.167
HR diff.	−0.002	0.011	72	−0.16	0.870
[Na^+^] loss	0.003	0.000	72	7.02	**<0.001**
[K^+^] loss	−0.006	0.011	72	−0.59	0.560
Weight diff.	0.003	0.000	72	7.02	**<0.001**

REML: restricted maximum likelihood. In bold: statistical significance; SE: standard error; DF: degrees of freedom; T: T-statistic; “Diff” indicates the difference in values across participants over the 9-day observation period. ST: diff. skin temperature difference; SBP diff.: systolic blood pressure difference; DBP diff.: diastolic blood pressure difference; HR diff.: heart rate difference; [Na^+^] loss: loss of sodium concentration; [K^+^] loss: loss of potassium concentration; Weight diff: weight difference.

**Table 4:** Coefficients of the mixed model adjusted by REML for sodium loss.

Fixed Effects	Estimator	SE	DF	T	*p*-Value
Constant	−5910.503	6133.068	72	−0.96	0.338
Day	36.725	49.847	72	0.74	0.464
ST diff.	−29.386	44.746	72	−0.66	0.514
SBP diff.	−51.541	27.545	72	−1.87	**0.065**
DBP diff.	41.935	19.002	72	2.21	**0.031**
HR diff.	−27.135	25.487	72	−1.06	0.291
[K^+^] loss	37.348	24.816	72	1.50	0.137
Sweat loss estim.	5.423	0.921	72	5.89	**<0.001**
Weight diff.	83.333	259.081	72	0.32	0.749

REML: restricted maximum likelihood. In bold: statistical significance; SE: standard error; DF: degrees of freedom; T: T-statistic; “Diff” indicates the difference in values across participants over the 9-day observation period. ST: diff. skin temperature difference; SBP diff.: systolic blood pressure difference; DBP diff.: diastolic blood pressure difference; HR diff.: heart rate difference; [K^+^] loss: loss of potassium concentration; Sweat loss estim.: sweat loss estimation; Weight diff: weight difference.

**Table 5:** Coefficients of the mixed model adjusted by REML for potassium loss.

Fixed Effects	Estimator	SE	DF	T	*p*-Value
Constant	1281.611	581.694	72	2.20	**0.031**
Day	1.755	5.170	72	0.34	0.735
ST diff.	8.404	4.456	72	1.89	**0.063**
SBP diff.	3.759	2.880	72	1.31	0.196
DBP diff.	−4.821	1.950	72	−2.47	**0.016**
HR diff.	3.118	2.618	72	1.19	0.238
[Na^+^] loss	0.058	0.010	72	5.96	**<0.001**
Sweat loss estim.	146.758	20.898	72	7.02	**<0.001**
Weight diff.	−4.139	2.434	72	−1.70	**0.093**

REML: restricted maximum likelihood. In bold: statistical significance; SE: standard error; DF: degrees of freedom; T: T-statistic; “Diff” indicates the difference in values across participants over the 9-day observation period. ST: diff. skin body temperature difference; SBP diff.: systolic blood pressure difference; DBP diff.: diastolic blood pressure difference; HR diff.: heart rate difference; [Na^+^] loss: loss of sodium concentration; Sweat loss estim.: sweat loss estimation; Weight diff: weight difference.

## Data Availability

The data presented in this study are openly available in the CORA Repositori de Dades de Recerca at https://doi.org/10.34810/data2652.
